# Competitive Stress Elicits Distinct Psychophysiological and Immunological Responses in Sub-Elite Water Polo Players

**DOI:** 10.3390/sports14060254

**Published:** 2026-06-22

**Authors:** Nika Nikousokhan Tayyar, Sara Naim, Antonella Strangio, Daniele Murgia, Luca Nanni, Daniele Saverino

**Affiliations:** 1 Department of Experimental Medicine, University of Genoa, 16132 Genova, Italy; nika.nikousokhan@studio.unibo.it (N.N.T.); antonella.strangio@unige.it (A.S.); 2 IRCCS ‘G. Gaslini’ Institute, 16132 Genova, Italy; 4201144@studenti.unige.it; 3 Department of Neurosciences, Rehabilitation, Ophthalmology, Genetics, Maternal and Child Health, University of Genoa, 16132 Genova, Italy; 4 Laboratory Medicine, IRCCS Azienda Ospedaliera Metropolitana, Plesso San Martino, 16132 Genova, Italyluca.nanni@aomliguria.it (L.N.); 5 Diagnostic Autoimmunology Laboratory, IRCCS Azienda Ospedaliera Metropolitana, Plesso San Martino, 16132 Genova, Italy

**Keywords:** competition stress, salivary biomarkers, cortisol reactivity, mucosal immunity (IgA), psychophysiological responses

## Abstract

**Objectives**: This study investigated the interplay between pre- and post-match physiological responses and subsequent emotional changes in male water polo players competing in the Italian Serie C league (third national level, sub-elite), focusing on differences between official championship (competitive) and non-competitive (training) settings. **Methods:** Sixteen male Italian Serie C water polo players participated. Salivary biomarkers (cortisol, immunoglobulin A (IgA), and uric acid) were measured, alongside psychological assessments of cognitive anxiety, somatic anxiety, and self-confidence. Measurements were taken before and after both training and competition matches. **Results:** A significant anticipatory rise in salivary cortisol was observed before competition matches compared to training, highlighting the psychological stress associated with competitive events. Post-match, cortisol levels remained elevated to a greater extent after competition. Salivary IgA levels decreased significantly following both training and competition, with a more pronounced reduction after official matches, and exhibited a negative correlation with cortisol. Salivary uric acid, a marker of oxidative stress, increased post-exercise and was significantly higher after competition. Players reported higher somatic and cognitive anxiety and lower self-confidence before competition compared to training, and pre-competition cortisol levels were positively correlated with both anxiety measures and negatively correlated with self-confidence. **Conclusions:** These findings highlight the distinct physiological and psychological responses elicited by competitive versus non-competitive settings in water polo, emphasizing the importance of considering the emotional context when monitoring athletes’ stress and recovery. The social meaning of competitive contexts may be embodied, impacting stress and immune responses.

## 1. Introduction

Water polo represents a high-intensity team sport characterized by significant metabolic, neuromuscular, and psychological demands [1]. The rigors of training and competition can lead to a spectrum of physiological states, ranging from acute fatigue to overreaching and, in severe cases, overtraining syndrome [2]. While strategic overreaching can enhance performance, overtraining is associated with prolonged performance decrements [3]. Therefore, meticulous monitoring of individual athletes is essential to evaluate training-induced effects on well-being and performance, facilitating the development of personalized recovery protocols. Individual biochemical parameters serve as valuable metrics for tailoring recovery strategies [4]. Physical and psychological stressors engage multiple physiological systems, including the hypothalamic–pituitary–adrenal axis, the sympathetic nervous system, and the immune system [5]. Corticotropin-releasing hormone (CRH) plays a central role in mediating these responses: activating the sympathetic nervous system, leading to an increased release of adrenaline, and enhancing cortisol production by stimulating the secretion of adrenocorticotropic hormone (ACTH) from the pituitary gland [6]. Exercise-induced stress elicits biological responses that vary according to intensity, duration, and the athlete’s training status [7]. Extended or intense physical activity elevates serum cortisol, increases adrenaline and cytokine release, and decreases immunoglobulin A (IgA) at both salivary and serum levels [8,9,10,11]. Traditional biochemical assessments typically involve invasive blood sampling, often met with athlete reluctance. However, research demonstrates a strong correlation between plasma and salivary biomarker concentrations, indicating that salivary analytes reflect systemic physiological changes [8,12,13]. Consequently, salivary analysis offers a non-invasive alternative for monitoring hormonal, immunological, and oxidative stress markers. This approach enhances athlete compliance and facilitates longitudinal monitoring, thus optimizing training and recovery interventions [8,12].

The relationship between endocrine activity and both mental and physical strain in athletes has been extensively studied, particularly when comparing individual and team sports [13]. In addition, cortisol levels are pivotal in the modulation of both systemic and neuroendocrine stress responses. Salivary cortisol quantification has been validated as a reliable proxy for assessing physiological and psychological stress induced by physical exercise [8,10,12,13,14]. Cortisol induces catabolic processes within skeletal muscle, impacting protein metabolism. This catabolic action facilitates the mobilization of free amino acids, which serve as supplementary substrates for oxidative phosphorylation during and post-exercise. This physiological response is crucial for homeostatic adaptation to stressors, such as those encountered during physical exertion [8,15].

Furthermore, salivary immunoglobulin A (IgA), a key component of mucosal immunity against exogenous pathogens within the upper respiratory tract, has been reported to exhibit a substantial reduction in football players following a competitive international match [16,17,18]. Notably, even a single training match can reduce salivary IgA, with considerable inter-individual variability [14,15].

Psychological adaptations, such as mood and emotional state, are shaped by the symbolic and socio-cultural context of competition [19]. The concept of embodiment—rooted in phenomenology and expanded in anthropology and psychology—posits that cognitive and emotional processes are deeply rooted in lived bodily experience and shaped by social context [20]. This perspective allows for the examination of physiological and psychological responses as interdependent phenomena, with the body serving as the site where social-symbolic perceptions, expectations, and self-confidence are manifested. Morgan and colleagues introduced the Profile of Mood States scale (POMS) in sports, demonstrating that elite athletes typically exhibit high vigor and lower anger, tension, fatigue, depression, and confusion scores compared to population norms [21]. However, athletes undergoing intense and strenuous training can shift the POMS profile characterized by reduced vigor and increased tension, depression, anger, fatigue, and confusion [22].

Personalized intervention in sport refers to the adaptation of training load, recovery strategies, and psychological preparation based on the individual athlete’s physiological, psychological, and contextual characteristics. This approach integrates biomarkers (e.g., cortisol, IgA), psychological profiling, and performance monitoring to optimize adaptation and reduce the risk of overtraining. Previous literature supports the effectiveness of individualized monitoring and recovery strategies in improving performance and athlete well-being [8,12]. The present study aimed to examine how emotions experienced in anticipation of a championship match influence neuroendocrine, oxidative, and immune changes in elite water polo players. These changes were compared with those observed before and after a standard training session (i.e., a friendly match), similar in intensity and duration. To achieve this, salivary cortisol, IgA, and uric acid levels before and after the various match sessions were measured. Finally, mood was assessed in the same context.

## 2. Materials and Methods

### 2.1. Participants

Participants were male water polo players competing in the Italian Serie C league (third tier of the Italian water polo system, representing a sub-elite competitive level). All athletes belonged to the same team and followed a standardized training program consisting of 4–5 weekly sessions (8–10 h/week) across a competitive season lasting approximately 8–9 months. All players participated in all match sessions; however, individual playing time and positional roles were not controlled and may have influenced physiological responses. Participants were instructed to maintain normal hydration and dietary habits; however, no strict control of sleep, nutrition, or supplementation was implemented, which should be considered when interpreting biomarker variability.

Sixteen players were selected: goalkeepers: n = 2, field players: n = 10, and center forwards: n = 4. Participant mean age was 23 ± 4 years (range 19–27).

### 2.2. Saliva Sample Collection and Analysis

All analytical tests in this study were performed on saliva rather than blood, due to the non-invasive nature of saliva collection and the autonomy it provides participants. Saliva can be collected directly by the athlete, without the need for medical personnel, and in environments such as swimming pools.

Saliva samples were collected before (PRE; 15–20 min prior) and after (POST; 15–20 min following) two training match sessions (SESSIONS 1 and 3) and two official league matches (SESSIONS 2 and 4). All sessions were scheduled between 7:00 PM and 9:00 PM to minimize circadian variability in biomarker levels.

Saliva samples were obtained using cotton swabs and saliva collecting tubes (SARSTEDT S.r.l., Trezzano sul Naviglio, Milano, Italy). The athletes were instructed to place the cotton swab into their mouths, under the tongue, for 2 min. The absence of blood contamination was checked with a salivary blood contamination kit (Salimetrics LLC, Cambridge, UK). The saliva collecting tubes were centrifuged at 3000 rev/min for 10 min at 4 °C. After collection, the swabs were placed in special tubes, stored in a refrigerated bag at 4 °C for a maximum of 2 h, and then were stored at −80 °C until they were assayed. To exclude inter-assay variance, all samples were thawed once and analyzed in duplicate in the same assay run. As stated above, participants refrained from consuming any food, drinking hot fluids, or brushing their teeth for two hours before their arrival. Upon their arrival, before starting the training session, participants were asked to remain seated for 15 min before providing their resting sample (PRE), and subsequently, they completed the training/match sessions. At the end of the training/match session, they were asked to remain in a relaxed position (e.g., seated or standing) for 15 min until the collection time point (POST). Concentrations of cortisol were assessed by enzyme immunoassay (EIA test, Pantex, Santa Monica, CA, USA), and IgA and uric acid (EMELCA Bioscience, Clinge, The Netherlands) were assessed via enzyme-linked immunosorbent assays (ELISA), following the manufacturers’ instructions, including sample incubation, washing steps, and optical density measurement using standard calibration curves. All samples were analyzed in duplicate, and the mean value was used for statistical analysis. For cortisol, the assay range was 0.1–300 μg/mL, and the sensitivity was 0.0392 μg/mL. For IgA, the range was 0.24–1000 μg/mL, and the sensitivity was <0.24 μg/mL. Finally, for uric acid, the range was 617.3–50,000 ng/dL (i.e., 7–16 μg/dL) with a sensitivity of 243.1 ng/dL. Uric acid concentrations were initially obtained in ng/dL according to the kit specifications and subsequently converted to μg/dL for reporting and statistical analysis. Raw values obtained in ng/dL were converted to μg/dL for clarity and to facilitate comparison with the existing literature. This unit conversion did not affect the relative differences between samples or the statistical outcomes. Intra-assay deviation was 6.3% for the EIA test and <10% for all ELISA kits. All measured biomarker concentrations fell within the assay detection range, confirming that the reported values were reliably quantified. When concentrations exceeded the upper limit of detection, samples were diluted accordingly using the manufacturer-provided sample diluent, and results were adjusted based on the dilution factor.

### 2.3. Cognitive and Somatic Anxiety, and Self-Confidence Assessment

The Competitive State Anxiety Inventory-2 (CSAI-2) was utilized [23] to assess athletes’ cognitive and somatic anxiety, as well as their self-confidence. Cognitive anxiety is understood as negative self-assessments and doubts regarding an athlete’s performance capabilities [24]. Somatic anxiety pertains to the athlete’s awareness of physiological symptoms of anxiety, such as muscle tension and elevated heart rate [25]. The CSAI-2 comprises 27 items, divided into three subscales: cognitive anxiety, somatic anxiety, and self-confidence, each containing 9 items. Responses are recorded on a 4-point Likert scale, yielding subscale scores ranging from 9 to 36. Higher scores on the cognitive and somatic anxiety subscales indicate greater anxiety levels, while higher scores on the self-confidence subscale reflect increased self-confidence. Sample items include statements such as “I am concerned about this competition” (cognitive anxiety), “My body feels tense” (somatic anxiety), and “I am feeling self-confident” (self-confidence). Previous studies reported Cronbach’s alpha coefficients of 0.89 for cognitive anxiety and 0.92 for both somatic anxiety and self-confidence [26]. In the present study, Cronbach’s alpha was not recalculated because the CSAI-2 is a widely validated instrument with established reliability and factorial validity across athletic populations [23,24,25,26,27]. Instead, we relied on previously published validation studies, including research conducted in comparable competitive sport contexts (e.g., tennis players), which supports the robustness of the instrument [23,24,25,26,27].

All participants were familiarized with the questionnaire, which was integrated into the team’s monitoring system by the coaching staff. Questionnaires were completed 15–20 min before and after each session. The factorial validity of the CSAI-2R was previously established by Cox et al. (2003) [27].

### 2.4. Statistical Analyses

Data distribution was assessed for normality using the Shapiro–Wilk test. Given the repeated-measures design, each participant contributed measurements across four sessions (two training matches and two competition matches), with PRE and POST values collected in each session.

To appropriately manage repeated observations and avoid inflation of degrees of freedom, values from sessions of the same type (training and competition matches) were averaged for each participant. This resulted in four conditions per subject: training PRE, training POST, competition PRE, and competition POST. Cortisol and uric acid concentrations were analyzed using a two-way repeated-measures ANOVA with SESSION (training vs. competition) and TIME (PRE vs. POST) as within-subject factors. IgA data were analyzed using the same approach. When appropriate, percentage changes (Δ%) were calculated as [(POST − PRE)/PRE × 100] for each condition, and comparisons between training and competition were performed using repeated-measures ANOVA. Effect sizes were calculated as partial eta squared (η^2^p) and interpreted according to conventional thresholds (small ≥ 0.01, medium ≥ 0.06, large ≥ 0.14). Spearman correlation coefficients (r) were used to assess relationships between psychological measures and biomarkers. The strength of correlations was interpreted as weak (r < 0.30), moderate (0.30–0.49), and strong (≥0.50). Session order was not included as a factor, as all athletes followed the same structured training and competition schedule. Given the balanced within-subject design and relatively small sample size, a repeated-measures ANOVA approach was preferred over mixed-effects modeling. Statistical analyses were performed using Prism10 (GraphPad Software, Boston, MA, USA). Statistical significance was set at *p* < 0.05. Data are presented as mean ± standard deviation.

## 3. Results

### 3.1. Salivary Cortisol Levels

Salivary cortisol levels (μg/mL) were analyzed using two-way repeated measures ANOVA, revealing significant effects of session type (training vs. competition) [F(1,15) = 2.021; *p* < 0.001], with a very large effect size (η^2^p = 0.76), suggesting a strong influence of experimental conditions on cortisol responses. A significant effect of time (PRE vs. POST) was observed [F(1,15) = 57.713; *p* < 0.001]. Finally, a significant interaction effect between session and time was observed (F(1,15) = 61.661; *p* < 0.001), with a very large effect size (η^2^p = 0.56), indicating that changes from PRE to POST differed significantly between training and competition matches.

**Figure 1:**
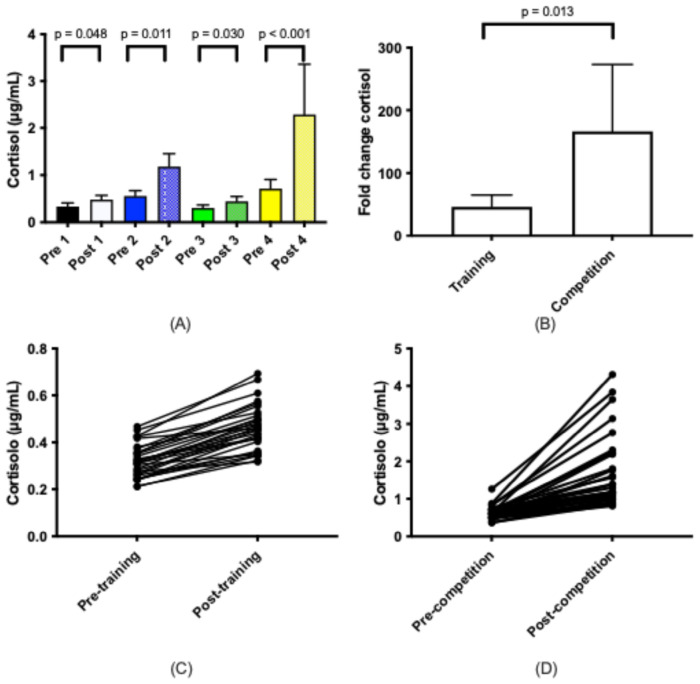
**Cortisol values and Δ% cortisol.** Panel (**A**) Cortisol values before (PRE) and after (POST) training matches (PRE 1 and 3, POST 1 and 3) and competition matches (PRE 2 and 4, POST 2 and 4). Panel (**B**) Δ% Cortisol of training (**left**) and competition matches (**right**). Panels (**C**,**D**) Pre- and post-cortisol values, respectively, for training and competitive sessions. Data are presented as means ± standard error.

Bonferroni post hoc tests indicated a significant increase in salivary cortisol from PRE to POST in all sessions: PRE 1: 0.382 ± 0.063 μg/mL, POST 1: 0.603 ± 0.100 μg/mL (*p* = 0.048); PRE 2: 0.570 ± 0.109 μg/mL, POST 2: 1.163 ± 0.273 μg/mL (*p* = 0.011); PRE 3: 0.317 ± 0.085 μg/mL, POST 3: 0.854 ± 0.343 μg/mL (*p* = 0.030); PRE 4: 0.801 ± 0.214 μg/mL, POST 4: 3.057 ± 0.807 μg/mL (*p* < 0.001) (Figure 1A).

Overall, competition matches elicited a greater increase in salivary cortisol than training matches: PRE-training: 0.349 ± 0.080 μg/mL, POST-training: 0.728 ± 0.276 μg/mL (*p* < 0.001); PRE-competition: 0.686 ± 0.203 μg/mL, POST-competition: 2.110 ± 1.138 μg/mL (*p* = 0.001) (Figure 1C,D).

The percentage change in cortisol (Δ% cortisol) was significantly greater during competition (201.2 ± 125.7%) compared to training (131.0 ± 138.3%; *p* = 0.013) (Figure 1B).

Overall, cortisol levels increased significantly from PRE to POST, with a greater increase observed during competition compared to training matches.

All analyses were conducted on participant-averaged values for each condition.

### 3.2. Salivary IgA Levels

Salivary IgA concentrations (μg/mL) were analyzed using two-way repeated measures ANOVA, revealing significant effects of session type [F(1,15) = 3.199; *p* = 0.007], with a large effect size (η^2^p = 0.29). A significant effect of time [F(1,15) = 58.710; *p* < 0.001], with an extremely large effect size (η^2^p = 0.74). Finally, a significant interaction between session and time was also found [F(1,15) = 124.241; *p* < 0.001], with an extremely large effect size (η^2^p = 0.72). The large SESSION × TIME interaction reflects the consistency of IgA suppression across repeated competitive sessions rather than an overestimation driven by outliers, as evidenced by comparable variance and directionality of responses across all sessions.

**Figure 2:**
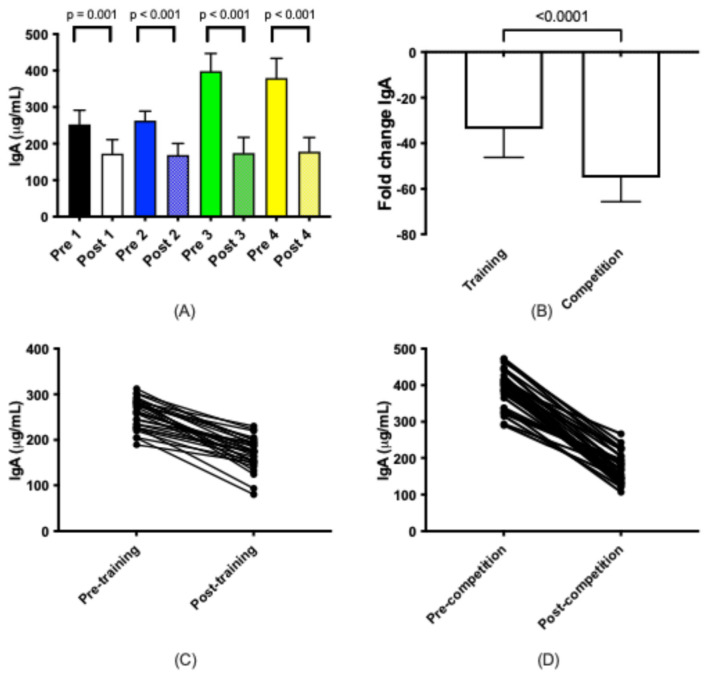
**IgA values and Δ% IgA.** Panel (**A**) IgA values before (PRE) and after (POST) training matches (PRE 1 and 3, POST 1 and 3) and competition matches (PRE 2 and 4, POST 2 and 4). Panel (**B**) Δ% IgA of training (**left**) and competition matches (**right**). Panels (**C**,**D**) Pre- and post-IgA values, respectively for training and competitive sessions. Data are presented as means ± standard error.

Bonferroni post hoc tests showed that IgA levels decreased significantly from PRE to POST in both training and competition matches: PRE 1: 249.40 ± 43.02 μg/mL, POST 1: 170.81 ± 27.82 μg/mL (*p* = 0.001); PRE 2: 271.33 ± 26.88 μg/mL, POST 2: 176.30 ± 25.53 μg/mL (*p* < 0.001); PRE 3: 375.82 ± 19.92 μg/mL, POST 3: 155.83 ± 18.92 μg/mL (*p* < 0.001); PRE 4: 380.44 ± 64.01 μg/mL, POST 4: 186.00 ± 27.82 μg/mL (*p* < 0.001) (Figure 2A).

**Figure 3:**
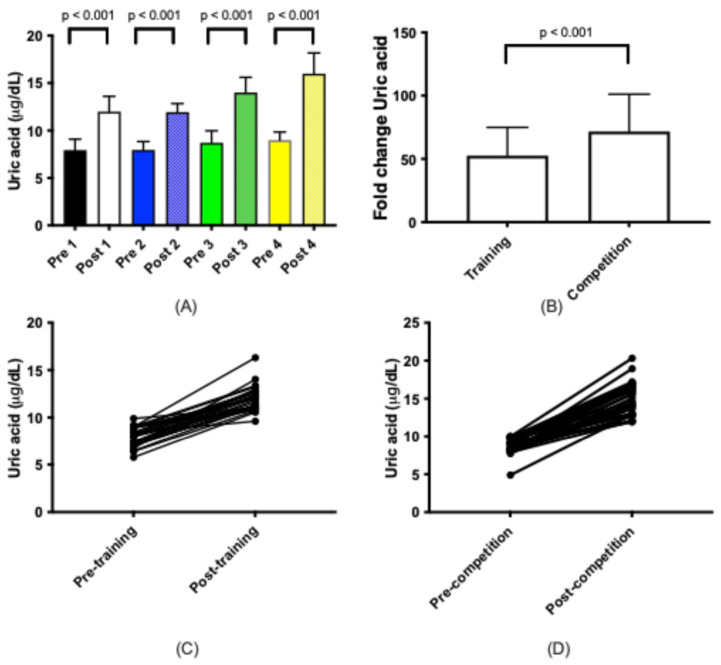
**Uric acid values and Δ% uric acid.** Panel (**A**) Uric acid values before (PRE) and after (POST) training matches (PRE 1 and 3, POST 1 and 3) and competition matches (PRE 2 and 4, POST 2 and 4). Panel (**B**) Δ% Uric acid of training (**left**) and competition matches (**right**). Panels (**C**,**D**) Pre- and post-uric acid values, respectively for training and competitive sessions. Data are presented as means ± standard error.

When pooling all training and all competition matches, IgA levels were consistently lower after activity: Training: 260.30 ± 36.45 μg/mL (PRE) vs. 173.50 ± 25.95 μg/mL (POST), *p* < 0.001; Competition: 378.10 ± 54.47 μg/mL (PRE) vs. 165.90 ± 29.17 μg/mL (POST), *p* < 0.001 (Figure 3C,D).

The percentage change in IgA (ΔIgA) was significantly greater during competition (−55.36 ± 9.70%) than during training (−32.60 ± 10.36%; *p* < 0.001) (Figure 2B).

Post hoc analyses confirmed a significant decrease in IgA levels from PRE to POST in both training and competition matches.

### 3.3. Salivary Uric Acid Levels

Salivary uric acid concentrations (expressed in μg/dL) were obtained from raw measurements expressed in ng/dL according to the kit specifications and subsequently converted to μg/dL for reporting. Based on this conversion, the observed values (approximately 7–16 μg/dL) correspond to 7000–16,000 ng/dL, which fall well within the assay detection range (617.3–50,000 ng/dL). Importantly, the observed PRE–POST differences (approximately 3–7 μg/dL, i.e., 3000–7000 ng/dL) exceed the assay sensitivity (243.1 ng/dL) by more than one order of magnitude, confirming that the assay is sufficiently sensitive and that the reported changes are analytically detectable.

Salivary uric acid levels were analyzed using two-way repeated measures ANOVA, revealing a significant effect of session type [F(1,15) = 107.70; *p* < 0.001], with a large effect size (η^2^p = 0.26), and a significant effect of time (PRE vs. POST) [F(1,15) = 46.70; *p* < 0.001], with a large effect size (η^2^p = 0.74). A significant SESSION × TIME interaction was also observed [F(1,15) = 136.0; *p* < 0.001; η^2^p = 0.74], indicating that changes from PRE to POST differed between training and competition matches.

Bonferroni post hoc tests showed a significant increase in uric acid from PRE to POST in all sessions: PRE 1: 7.828 ± 1.372 μg/dL, POST 1: 11.810 ± 1.107 μg/dL (*p* < 0.001); PRE 2: 7.783 ± 1.020 μg/dL, POST 2: 11.470 ± 0.725 μg/dL (*p* < 0.001); PRE 3: 8.614 ± 1.706 μg/dL, POST 3: 13.940 ± 1.656 μg/dL (*p* < 0.001); PRE 4: 8.992 ± 0.848 μg/dL, POST 4: 16.080 ± 1.586 μg/dL (*p* < 0.001) (Figure 3A).

**Figure 4:**
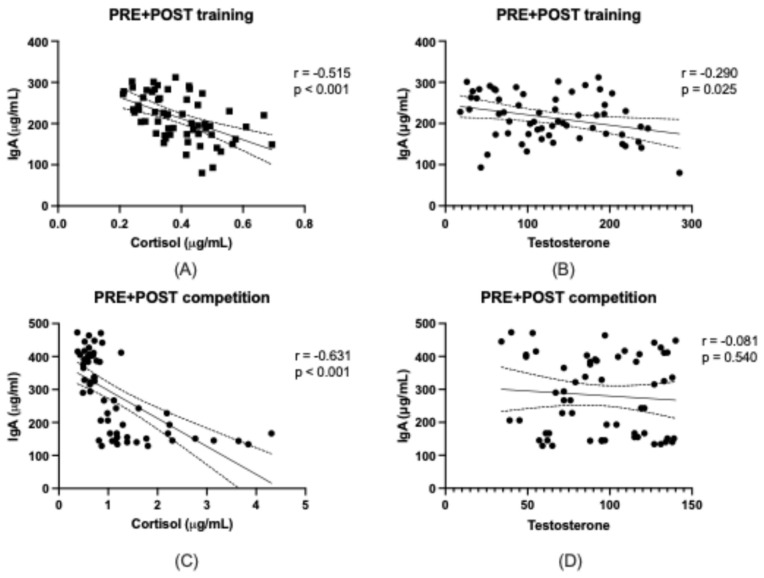
**Relationship among Cortisol, IgA, and uric acid values after training and competition matches.** A significant negative relationship appeared between cortisol and IgA values during PRE- and POST-training matches (panels (**A**) and (**B**), respectively). On the contrary, a positive relationship was observed between cortisol and uric acid in the same experimental conditions (panels (**C**,**D**)).

When pooling sessions, uric acid was higher after activity: PRE-training: 7.805 ± 1.168 μg/dL, POST-training: 11.640 ± 0.972 μg/dL (*p* < 0.001); PRE-competition: 8.803 ± 1.316 μg/dL, POST-competition: 15.010 ± 1.917 μg/dL (*p* < 0.001) (Figure 4C,D).

Finally, the overall effect of the type of activity (training vs. competition) showed a significant increase in salivary uric acid levels, highlighting the role of the match type in determining a significant increase in uric acid production (POST-training vs. POST competition *p* < 0.001). Of interest, uric acid levels did not appear to be influenced by water polo players waiting for the game (PRE-training vs. PRE-competition *p* = 0.171). These findings suggest that uric acid responses are primarily driven by the physical demands of match play rather than anticipatory stress alone.

Furthermore, comparing the Δ% uric acid values between the two sessions, “training” and “competition”, a significant difference emerged (Figure 3B). Players during the training matches were characterized by a Δ% uric acid of 52.510 ± 26.80%, while during the competition matches by 74.50 ± 34.96%, *p* = 0.008.

Overall, these findings indicate that salivary uric acid increases following exercise and is modulated by match context, with larger responses observed during competition. Given that all values lie within the assay detection range and exceed the analytical sensitivity threshold, these changes can be reliably detected and interpreted as reflecting exercise-related oxidative stress responses.

### 3.4. Relationships Among Cortisol, IgA, and Uric Acid

A significant negative relationship between cortisol and IgA values during PRE- and POST-training matches was evident: the higher the PRE- or POST cortisol values, the lower the PRE- or POST IgA values (r = −0.454, *p* = 0.009). Similar results were obtained comparing cortisol and IgA values PRE or POST competition matches (r = −0.541, *p* = 0.001) (Figure 4A,B).

Conversely, a positive relationship between cortisol and uric acid was observed (Figure 4C,D), both during training matches and during competition matches (r = 0.534, *p* = 0.009) than during the competition matches (r = 0.515, *p* < 0.001).

### 3.5. Psychological Measures

CSAI-2 questionnaire results are summarized in Table 1. Significant differences were observed between training and competition matches for both pre- and post-session somatic and cognitive anxiety, with higher values during competition.

Somatic anxiety differed significantly between pre- and post-session for both training (*p* < 0.001) and competition (*p* = 0.002). Cognitive anxiety also differed significantly between pre- and post-session for training (*p* = 0.003) and competition (*p* = 0.005).

Self-confidence scores were significantly lower before than after both training and competition (*p* < 0.001 for both). Furthermore, self-confidence was significantly lower during competition matches compared to training (*p* < 0.001).

### 3.6. Correlations Among Psychological Parameters and Cortisol

**Figure 5:**
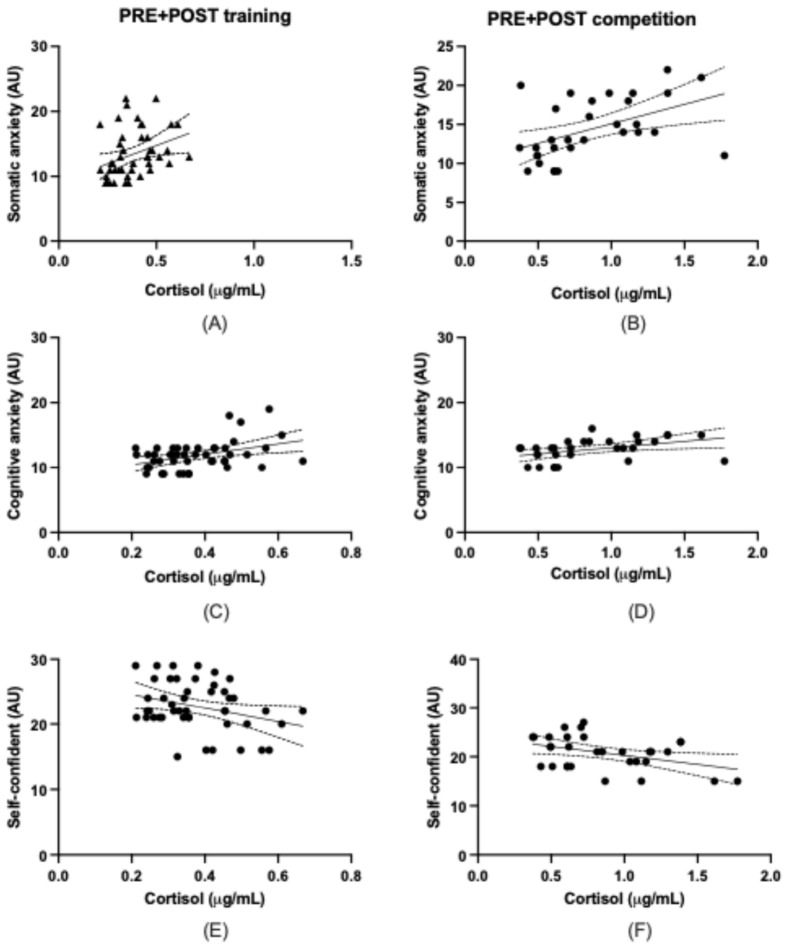
**Correlations among psychological parameters and cortisol.** Positive correlations between salivary cortisol concentrations and (**A**,**B**) somatic anxiety, (**C**,**D**) cognitive anxiety, and an inverse correlation between cortisol levels and self-confidence (**E**,**F**) in elite water polo players, measured before and after training and competition matches. AU = Arbitrary Units.

Correlation analyses were conducted to examine the relationship between CSAI-2 components and salivary cortisol concentrations measured before and after different sessions, both in training and competitive matches (Figure 5). Cortisol concentrations were significantly correlated with CSAI-2 somatic anxiety in both training and competitive sessions (r = 0.548, *p* < 0.001, and r = 0.572, *p* < 0.001). Similarly, cortisol was positively correlated with CSAI-2 cognitive anxiety in both training (r = 0.548, *p* < 0.001) and competition (r = 0.524, *p* < 0.001) sessions.

Cortisol levels were negatively correlated with CSAI-2 self-confidence during training matches (r = −0.473, *p* = 0.005). During competition matches, a weaker yet statistically significant negative association was also observed (r = −0.288, *p* = 0.026), suggesting that higher cortisol concentrations are consistently associated with lower perceived self-confidence across both conditions, albeit with reduced strength under competitive conditions.

## 4. Discussion

It is widely established that challenges encountered during one’s working life (i.e., unemployment, low salary, particularly demanding work activities) can be associated with an increased risk of developing health issues [28,29]. Similarly, prolonged and intense physical activity, such as that induced by official sports competitions, elicits stress levels and influences the immune responses of athletes differently compared to non-competitive activities [28,29,30,31,32]. This confirms the relationship between hormonal and immunological alterations and aggressive and offensive behaviours expressed during the game [30,31,32].

The present research investigates the influence of emotional processes that characterize the anticipation phase of training/competition (“friendly/official”) matches on specific hormonal, immunological, and oxidative stress responses that manifest during a match in a team of professional water polo players. The objective was to evaluate the stress response in elite water polo athletes during a training and a competitive session. Furthermore, under the same conditions, the effect on the mucosal immune response was evaluated by measuring salivary IgA levels and the state of oxidative stress by assessing uric acid levels.

Before the competition match, salivary cortisol levels increased more than those observed before the training session. These results are consistent with various studies indicating that anticipatory psychological stress associated with competition triggers cortisol secretion [8,10,12,13,14,17,33]. In both cases, an increase in cortisol levels is evident; however, in the case of a formal competition, the rise is significantly higher. Elevating cortisol concentrations in the pre-competitive phase plays a salient role in activating competitive motivation and mobilizing physiological resources necessary to prepare the athlete for the impending event [34]. Thus, the anticipatory increase in salivary cortisol occurs before sports competition, characteristic of athletes under psychosocial and physical stress conditions [34]. Cortisol levels were also elevated after the competition compared to the training session. This substantial increase in cortisol and, consequently, the decrease in IgA, can be interpreted through the lens of embodiment. This response is not merely an objective physiological measurement but a manifestation of symbolic perception and social relations: the body reflects the different meanings and social dynamics between a friendly match and a formal competition in the form of physiological changes following the event. Furthermore, an increase in cortisol levels was observed at the end of training, albeit of lesser magnitude, during the resting period (pre-training match). In summary, a significant variation in cortisol levels was also reported before and after training and on the day of competition. These data are consistent with previously reported findings [34,35], highlighting the functional role of cortisol elevation, which, under acute stress conditions, may enhance cognitive processes such as attention and information processing [34,35,36,37,38,39,40].

Several scientific studies show that hormone levels in athletes (like testosterone and cortisol) can change depending on how skilled they are in their sport, whether they are male or female, their role on the team, and how well they play [14,34,35,36,37,38,39,40]. Often, top-level athletes have more testosterone (a hormone that helps build muscles) and less cortisol (a hormone linked to stress), while less experienced athletes might have the opposite reaction [14,34,35,36,37,38,39,40]. In water polo, each player’s time in the water can vary greatly. Therefore, factors such as playing duration, specific role within the team, and physical workload can differentially influence athletes’ stress responses [14,41,42,43]. Changes in hormones after exercise can indicate if an athlete is training too hard (a phase called “overreaching” or “overtraining”) [40]. The use of salivary biomarkers provides a reliable and non-invasive approach for assessing physiological responses in athletes, as salivary concentrations have been shown to reflect systemic changes comparable to those observed in blood [10,13]. These changes are consistent with physiological responses to acute exercise and fall within ranges commonly reported in athletic populations in the literature, supporting their interpretation as transient and adaptive rather than indicative of pathological stress [8,9,10,11,12,13,14,17,41]. However, reference ranges for salivary biomarkers can vary depending on methodological and individual factors, and results should be interpreted within the specific experimental context [12,13,14,15]. It is interesting to note that cortisol levels were higher in the later stages of training or competitive matches compared to the first, perhaps because their bodies were preparing for the upcoming competitions. In addition, similar patterns have been reported in team sports such as soccer, where competitive matches are associated with significant increases in stress-related hormones, particularly cortisol, reflecting the psychological and physiological demands of competition [10,13,29,30]. Notably, studies in soccer players have shown elevated pre-competition cortisol levels linked to anticipatory stress, as well as sustained post-match increases [10,13,29,30]. These findings are consistent with the present results, which demonstrate both anticipatory and post-competition increases in cortisol in water polo players. This cross-sport consistency supports the notion that cortisol reactivity represents a generalizable marker of competitive stress in team sports. Intense physical activity has been shown to induce significant alterations in immune function, including modulation of the body’s defense mechanisms against illness [8,11,42,43,44,45,46]. Mucosal immunity, primarily mediated by IgA, plays a critical role as the first line of defense against pathogens entering the upper respiratory tract and is particularly sensitive to exercise-induced stress [42,43,44,45,46]. Reduced salivary IgA levels have been associated with an increased risk of upper respiratory tract infections in athletes, particularly during periods of intense training or competition [42,43,44,45,46]. In addition, several studies have indicated that in the saliva of athletes participating in team sports, IgA levels fluctuate depending on the season, the significance of a match, and the intensity or type of physical exertion [42,43,44,45,46]. Furthermore, the mucosal immune response may differ between competitive athletes and non-athletes, likely associated with the varying levels of physiological stress experienced [47]. These differences in immune responses can be interpreted through the lens of embodiment. From this perspective, the psychological perception of stress, particularly the symbolic meaning attributed to competition, may modulate physiological responses differently in competitive compared to non-competitive contexts. In the present study, IgA levels decreased following both training and competition matches, with a significantly greater reduction observed after official competition. This finding suggests that competitive stress may exacerbate exercise-induced mucosal immunosuppression. These results are consistent with previous literature indicating that intense physical activity can induce significant alterations in immune function, particularly in mucosal immunity mediated by immunoglobulin A (IgA), which serves as a primary defense against pathogens entering the upper respiratory tract [8,11,42,43,44,45,46]. In addition, IgA concentrations were inversely correlated with cortisol levels in both training and competitive contexts. This relationship supports the hypothesis that stress-related glucocorticoid activation may contribute to transient immune suppression [8,44,45,46,47,48,49]. However, previous studies have reported heterogeneous results, with some investigations showing a clear inverse relationship between cortisol and IgA, while others report weaker or non-significant associations [44,45,46,47,48,49]. Such variability may be explained by differences in study design, including the type and intensity of exercise, the timing of sample collection, and the characteristics of the studied populations (e.g., training status, sport discipline, and individual variability) [14,44,45,46,47,48,49]. In the present study, the observed inverse correlation between cortisol and IgA is consistent with research conducted in team sports under competitive conditions, suggesting that acute psychophysiological stress may transiently suppress mucosal immunity. The robustness of the IgA response is supported by the consistent direction and magnitude of suppression observed across all competitive sessions, indicating a stable and reproducible physiological response across repeated conditions. These findings are consistent with previous studies reporting exercise-induced reductions in salivary IgA in team sport athletes [37,38,39]. However, this correlation is not always straightforward. Some studies highlight that a clear link between salivary IgA and cortisol is not always evident [44,45,46,47,48,49]. Therefore, the relationship between IgA and cortisol may depend on multiple factors, including exercise intensity and duration, sport-specific demands, and individual variability, all of which have been shown to influence endocrine and mucosal immune responses in athletes [14,15,44,45,46,47,48,49]. These factors likely contribute to the variability observed across different studies. Further research is needed to better understand the relationship between IgA and salivary cortisol.

While regular/moderate physical exercise or recreational sports activity significantly improve redox parameters and confer numerous health benefits, intense exercise/competitive sport can generate an excess of oxidative stress, potentially leading to damage to the organism [8,11,50]. We evaluated oxidative stress using salivary uric acid as a biomarker, given that the oxidation of salivary proteins represents the first line of defense against free radicals in oral tissues [8]. Our observations indicated that salivary uric acid levels increased post-exercise and were significantly higher in pre-/post-competition matches compared to pre-/post-training matches. Uric acid levels were positively correlated with salivary cortisol concentrations, with a moderate positive association (r ≈ 0.51–0.53, *p* < 0.01), according to standard correlation thresholds. This finding suggests a coordinated response between oxidative stress and neuroendocrine activation, indicating that exercise-induced increases in cortisol are accompanied by elevated levels of uric acid, a marker of oxidative stress. It is known that cortisol increases concurrently with elevated oxidative stress following acute exercise [8,50]. Finally, the very large effect sizes observed for session-related conditions suggest that competitive environments exert a dominant influence on physiological responses.

Regarding the anxiety components, we found significant differences when comparing training and competitive sessions. Specifically, players exhibited greater somatic and cognitive state anxiety and lower self-confidence in the pre-competitive phase compared to the training phase. These results are consistent with other studies that have demonstrated significant game effects on cognitive and somatic state anxiety, as well as self-confidence in various sports [51,52,53]. Somatic state anxiety is related to the perception of physiological responses to psychological stress [35,54]. Additionally, cognitive anxiety, which is associated with fear about the consequences of failure [55,56], was reported to be higher in competitive sessions compared to training matches. These findings are consistent with recent research in other sports, suggesting the anxiogenic nature of sports competitions [34,50,51,52,53,54,55,56,57]. Moreover, the high-anxiety state observed was concurrent with the anticipatory response of salivary cortisol, confirming the previously described relationship between cortisol and competition-related negative mood [34,50,51,52,53,54,55,56,57]. Therefore, cortisol concentrations before competitive sessions were significantly and positively correlated with cognitive and somatic anxiety scores. Thus, for the self-confidence parameter, consistent with other studies [34,50,51,52,53,54,55,56,57], players showed higher values in the pre-training compared to the competitive sessions, further confirming the psychological disturbances induced by competitive situations, which were detected early through the reported physiological anticipatory responses. Finally, elevated pre-competition anxiety levels and associated cortisol increases, contrasted with lower self-confidence, suggest a potential psychological state that could negatively impact decision-making processes during critical championship water polo matches compared to training scenarios. In addition, the elevated levels of pre-competition anxiety and the associated increase in cortisol, coupled with lower self-esteem, suggest a potential psychological state that may negatively affect decision-making processes during crucial water polo matches, as opposed to training scenarios. The importance of the concept of embodiment in psychological experiences is supported by the findings of this study, which highlight a strong relationship between anxiety, cortisol levels, and decision-making processes, reflecting the integration of psychological and physiological responses. This relationship becomes evident through the analysis of specific biomarkers, namely cortisol, IgA, and uric acid, which collectively reflect the interaction between psychological stress, neuroendocrine activation, and immune function. Altogether, these findings contribute to a deeper understanding of the human condition, showing how objective data and subjective experiences are deeply interconnected and, when examined together, can offer a more comprehensive perspective in behavioral and physiological research.

The relationship between cortisol, anxiety, and decision-making in sports is complex and multifactorial, involving dynamic interactions between neuroendocrine activation and psychological processes, which together influence cognitive performance, attention, and behavioral responses during competition [34,50,51,52,53,54,55,56,57]. This framework has been consistently reported in studies examining stress responses in athletes. During sports competitions, cortisol levels increase in response to anticipatory stress, reflecting activation of the hypothalamic–pituitary–adrenal axis and preparing the athlete for the psychological and physiological demands of competition. This response has been consistently documented across various sports contexts, supporting its role as a general marker of competitive stress [8,10,12,13,14,17,34]. Somatic anxiety is related to the perception of physiological responses to psychological stress, such as increased heart rate and muscle tension [32,34]. Cognitive anxiety refers to negative expectations and concerns about performance outcomes, including fear of the consequences of failure, as defined in the sport anxiety literature [23,24]. Moderate levels of cortisol can have a positive effect on sports performance, improving cognitive processes and attention control [34]. However, excessively high or low levels of cortisol can have debilitating effects, reducing the inhibition of irrelevant stimuli for the task and compromising performance [34]. On the other hand, elevated cortisol levels can lead to a decrease in muscle mass, compromised recovery, and reduced energy levels, negatively affecting sports performance [34].

Anxiety can negatively influence decision-making in sports, as it can reduce the ability to concentrate and increase the tendency to make impulsive decisions [57]. Cognitive anxiety can lead to greater concern about the consequences of failure, negatively affecting the ability to make quick and accurate decisions [57]. Managing anxiety through relaxation techniques and psychological interventions can improve decision-making, helping athletes maintain calm and concentration during competitions [57].

## 5. Conclusions

This study highlights the significant physiological and psychological impact of competitive matches compared to training matches in sub-elite water polo players. The anticipation and execution of a championship game elicited a more pronounced stress response, characterized by increased cortisol and uric acid levels and a greater reduction in salivary IgA. These physiological changes were associated with altered psychological states, as higher levels of cognitive and somatic anxiety and lower self-confidence were observed before competition, together with increased cortisol concentrations. This supports the close relationship between neuroendocrine stress responses and perceived psychological states in competitive conditions. The observed correlations further reinforce the link between stress hormones, anxiety, and immune function. From a theoretical perspective, these findings can be interpreted through the lens of embodiment, suggesting that athletes’ perceptions of the competitive context are reflected in measurable physiological responses [19,20]. However, important contextual variables—such as opponent level, match outcome (e.g., victory or defeat), and competitive scenario—were not controlled and may have influenced the observed responses, particularly in the post-competition phase [14,41,42,43]. From an applied perspective, these results emphasize the importance of integrating physiological and psychological monitoring to better understand athletes’ responses to competitive stress. Personalized interventions—including stress management strategies, recovery optimization, and psychological preparation—may help optimize performance and reduce maladaptive stress responses. Future research involving larger and more diverse samples across different competitive levels is needed to confirm and extend these findings.

## 6. Limitations of the Study

Several limitations should be considered when interpreting these findings. First, the relatively small sample size may limit the statistical power and generalizability of the results. Second, the study included only male athletes, which may restrict the applicability of the findings to female populations, given potential sex-related differences in hormonal and psychological responses. Additionally, participants were recruited from a single team competing at a sub-elite level, which may limit the extrapolation of results to higher-level athletes or different competitive contexts. Factors such as playing position, individual playing time, and match-specific workload were not controlled and may have influenced physiological and psychological responses. Furthermore, variables such as sleep, nutrition, hydration status, and supplementation were not strictly monitored, which may have contributed to inter-individual variability in biomarker responses. Taken together, these limitations suggest that caution is warranted when generalizing these findings to broader athletic populations. Further research involving larger and more diverse samples across different competitive levels is needed to confirm and extend these findings.

## Figures and Tables

**Table 1:** Somatic, cognitive, and self-confidence score values obtained during training and competitive situations. Data presented as mean ± Standard deviation.

	Somatic Anxiety		Cognitive Anxiety		Self-Confident	
Condition	Pre	Post	Pre	Post	Pre	Post
Training	11.63 ± 3.22	16.00 ± 3.63	11.06 ± 1.57	13.19 ± 2.76	25.13 ± 3.14	20.13 ± 3.13
Competition	12.38 ± 3.46	16.50 ± 3.06	11.88 ± 1.41	13.56 ± 1.54	22.19 ± 3.23	19.13 ± 3.12

## Data Availability

Data will be made available upon reasonable request.
